# Comparison of Medical Management versus Parathyroidectomy in Patients with Mild Primary Hyperparathyroidism: A Meta-Analysis

**DOI:** 10.3390/cancers15123085

**Published:** 2023-06-07

**Authors:** Katherine A. Cironi, Peter P. Issa, Aaron L. Albuck, Christina McCarthy, Leely Rezvani, Mohammad Hussein, Xinyi Luo, Mohamed Shama, Eman Toraih, Emad Kandil

**Affiliations:** 1 School of Medicine, Tulane University, New Orleans, LA 70112, USA; 2 School of Medicine, Louisiana State University, New Orleans, LA 70112, USA; 3 Genetics Unit, Department of Histology and Cell Biology, Faculty of Medicine, Suez Canal University, Ismailia 41511, Egypt

**Keywords:** mild primary hyperparathyroidism, parathyroidectomy, meta-analysis

## Abstract

**Simple Summary:**

Patients undergoing medical treatment for mild pHPT maintain higher calcium and PTH levels. Patients managed medically had worse BMD outcomes. Considering this, and the superior biochemical and musculoskeletal outcomes which our study elucidates, we recommend that parathyroidectomy should be considered a superior treatment option compared to medical management for most patients with mild pHPT.

**Abstract:**

Background: Parathyroidectomy is the definitive cure for patients with primary hyperparathyroidism (pHPT) and has an annual prevalence of 0.2–1% in the United States. Some patients with mild disease are medically managed effectively using calcium-lowering medications and drugs against complications such as osteoporosis; however, many maintain a persistently high calcium level that negatively impacts their skeletal, renal, and psychogenic systems over the long term. This meta-analysis aims to compare the outcomes of medical management versus parathyroidectomy in patients with mild pHPT. Study Design: This meta-analysis was performed in accordance with the Preferred Reporting Items for Systematic Reviews and Meta-Analyses (PRISMA) guidelines using PubMed, Embase, and Web of Science by two teams of investigators. Analysis was run using R packages. Results: A total of 12 publications including seven randomized control, two prospective, and three retrospective trials with a total of 1346 patients were included for analysis. The average follow-up for all patients was 41 ± 23.8 months. Demographics, pre-treatment calcium, PTH, and bone mineral density (BMD) were similar between the medical (*N* = 632) and surgical (*N* = 714) cohorts. Post-treatment calcium and PTH levels were significantly higher in the medical cohort (10.46 vs. 9.39, *p* < 0.01), (106.14 vs. 43.25, *p* = 0.001), respectively. Interestingly, the post-treatment PTH in the medical cohort increased when compared to pre-treatment (83.84 to 106.14). Patients in the medical cohort had lower BMD in lumbar (0.48 g/cm^2^; OR = 0.42, 95% CI = 0.21, 0.83), femoral (0.48; OR = 0.42, 95% CI = 0.29, 0.61), and hip (0.61; OR = 0.33, 95% CI = 0.13, 0.85). Incidences of fracture, nephrolithiasis, cardiovascular death, or overall mortality were not significantly different between the cohorts. Conclusions: The present study is the most comprehensive meta-analysis on mild pHPT to date. Our findings reflect that parathyroidectomy is the superior option in the treatment of mild pHPT patients as opposed to medical management.

## 1. Introduction

Primary hyperparathyroidism (pHPT) is an endocrine disorder caused by inappropriate excess production of parathyroid hormone (PTH) with an annual prevalence ranging between 0.2% and 1% within the United States, according to a 2010 study [1]. Long-term consequences of hypercalcemia may manifest clinically with a plethora of long-term repercussions. These manifestations can include musculoskeletal diseases such as osteoporosis vertebral fractures, muscular weakness, and bone pain; renal complications such as nephrolithiasis, hypercalciuria, and polyuria; cardiovascular conditions including arrhythmia and high blood pressure; and psychiatric diseases including fatigue, depression, and anorexia [2,3,4]. Parathyroidectomy is the definitive cure for patients with pHPT [5].

Within the cohort of patients diagnosed with pHPT, a considerable subset exhibits a milder form of pHPT. This milder form of the disease is characterized by hypercalcemia accompanied by both upper-normal or elevated PTH, albeit without any discernible symptoms. The prevalence of this milder variant of pHPT is estimated to range between 5% and 28% of all pHPT patients [6,7,8]. Therapeutic strategies for these patients encompass both surgical interventions, in the form of parathyroidectomy, and conservative medical management. Medical management typically involves the administration of calcium-lowering medications, such as calcimimetics, along with bisphosphonates to counteract bone demineralization. Parathyroidectomy typically focuses on the removal of a single adenoma, given that 80–85% of pHPT cases can be attributed to this factor [9]. While the conservative management strategy does bypass the inherent risks associated with parathyroidectomy, it does come with its own set of challenges. Specifically, patients often continue to exhibit upper-normal or high calcium levels, which can negatively impact their health in the long run.

In general, the incidence of mild pHPT has been steadily rising as methods of detection both improve and become more widespread. This increase has gone hand in hand with an increase in the number of parathyroidectomies performed in recent years [10]. This trend seems to be universal, as a recent study from Hong Kong found a seven-fold increase in operative pHPT over the past decade [11], whereas a study from Denmark discovered a five-fold increase in the diagnosis of pHPT over a similar timespan [12]. Amid this increase in the diagnosis of mild pHPT, there is particular interest in the optimal treatment modality for the disease. A recent 2022 randomized controlled trial (RCT) has indicated that patients with mild pHPT managed either through medical or surgical means had similar morbidity and mortality rates with no significant disparity between the two cohorts [13]. Our current study aims to expand on this narrative, updating the existing literature with the most recent meta-analysis on this topic by Anagnostis et al. (2021). The aim of the present study is, therefore, to integrate recently published works to systematically compare the outcomes of non-operative management and parathyroidectomy in patients with mild pHPT [14]. This comparison, we believe, will shed further light on the optimal treatment strategy for this patient population.

## 2. Methods

### 2.1. Study Design and Search Strategy

This comprehensive meta-analysis was meticulously designed and conducted in accordance with the authoritative guidelines put forth by the Preferred Reporting Items for Systematic Reviews and Meta-Analyses (PRISMA) guidelines [15]. We initiated a month-long systematic literature exploration in June 2022, aiming to identify and collate all published studies that offer a comparative analysis of the management strategies employed for mild pHPT. Our search was broad and inclusive, utilizing search engines such as PubMed, Embase, and Web of Science. We applied an exhaustive list of search terms encompassing various aspects of the condition and its management. These terms included but were not limited to (primary hyperparathyroid*) AND (mild OR uncomplicate* OR asymptomatic* OR nonspecific* OR “non-specific”) AND (surgery* OR surgical* OR parathyroidectomy* OR “minimally invasive” OR explor*) AND (surveillance OR “medical management” OR “medical treatment” OR pharmaceutical OR “non-surgical” OR observation) AND (fracture OR skeletal OR bone OR renal OR kidney). To ensure no relevant studies were overlooked, we also scrutinized the reference lists of pertinent articles.

### 2.2. Study Selection

We included only peer-reviewed, full-text original articles that scrutinized the management strategies for mild pHPT in which mild is defined as hypercalcemia accompanied by either upper-normal or elevated PTH, without any discernible symptoms. We sought studies that profiled patients with mild pHPT, defined as asymptomatic individuals with elevated calcium levels, and discussed relevant outcomes. We did not implement specific biochemical thresholds when considering study populations for inclusion. The cohort managed medically comprised patients who were either observed or prescribed calcium-lowering or modifying medications such as calcimimetics and bisphosphonates. We were particularly interested in outcomes including calcium and PTH laboratory values, instances of nephrolithiasis or other renal implications, and bone mineral density (BMD) or indications of musculoskeletal health. We imposed no time constraints on our study screening process, and both RCTs and observational studies were incorporated into our meta-analysis. We excluded reviews, case reports, case series, editorials, letters to the editor, preprints, and published abstracts.

### 2.3. Data Extraction

Two teams of investigators proceeded with study selection and data extraction. The two teams of authors screened the articles by title, abstract, and, if necessary, full manuscript independently. Data extraction commenced utilizing a pre-designed data extraction sheet provided by statisticians. Parameters extracted included article authors, year published, title, institution, type of study, study duration, publication journal, and relevant demographics, including sample size, age, gender, and body mass index (BMI). Preoperative and postoperative calcium and PTH laboratory measurements were recorded. BMD reports at the lumbar spine, femoral neck, hip, forearm, and total body were collected at baseline and the final available follow-up. Incidences of new fractures and nephrolithiasis were recorded. Finally, the number of patients who underwent parathyroidectomy while under observational management as well as overall mortality were collected. When encountered, patients who were initially assigned to the observational group but underwent parathyroidectomy at a later point in time were included in the observational group consistent with the intention to treat notion. Patients who were initially intended to be treated by parathyroidectomy but received medical management preoperatively were included in the parathyroidectomy treatment cohort.

### 2.4. Statistical Analysis

All data analyses were performed using RStudio Build. First, a single-arm meta-analysis for laboratory tests was performed. Mean raw data (MRAW) and arcsine transformed proportion and 95% confidence intervals (CI) were reported for the estimated pooled results from studies. For pairwise comparison, estimates of standardized mean difference (SMD) served as quantitative measures of the strength of evidence, which were then converted to the odds ratio (OR) with a 95% confidence interval (CI) for better interpretation by clinical domains. For categorical outcomes, we computed either the relative risk (RR) or OR with a 95% CI. The OR is a measure of association between an exposure and an outcome, providing a comparative analysis of the odds of an event occurring in one group versus the odds of it occurring in another group. Relative risk is a ratio of the probability of an event occurring in the exposed group versus a non-exposed group.

Initially, we used a fixed effects model for the meta-analysis, which assumes that the true effect size is the same in all studies. This model provides a weighted average of the effect sizes, where the weight assigned to each study is determined by the study’s size and variance. However, when significant heterogeneity was detected among the study results (I^2^ > 50%), we pivoted to a random effects model instead. This model assumes that the true effect size varies from study to study and takes into account the variability between studies when estimating the overall effect size and uncertainty. This approach was deemed more appropriate in the presence of considerable heterogeneity, as it provides a more conservative estimate that widens the confidence intervals.

## 3. Results

### 3.1. Study Characteristics

**Figure 1:**
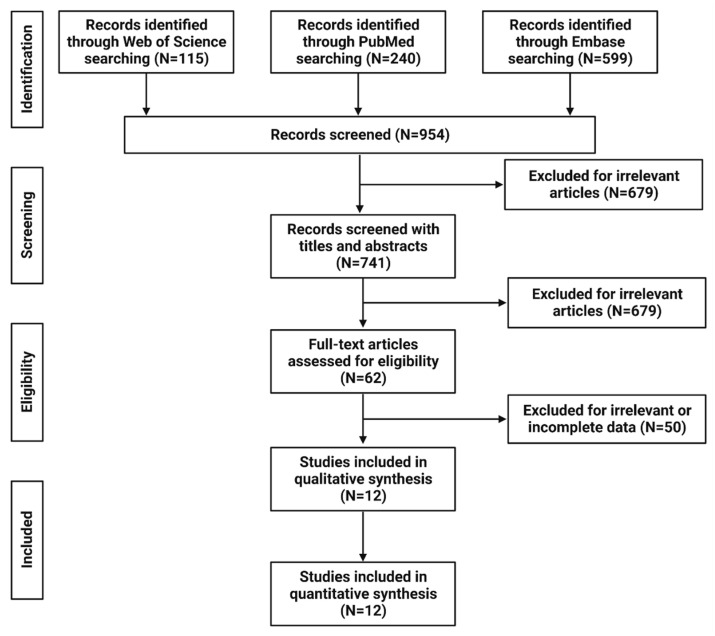
Study selection workflow.

An extensive literature search culminated in the identification of 954 published articles. Upon further inspection, 213 of these were detected to be duplicates, leaving us with 741 unique articles. These articles were then subjected to a detailed full-text eligibility criteria screening, which resulted in 62 articles being shortlisted for further analysis. A more stringent review process based on our inclusion criteria led to the selection of 12 articles, which were deemed suitable for a comprehensive quantitative analysis. The workflow detailing the selection of these studies is illustrated in Figure 1.

Though one research group published multiple studies (Bollerslev et al. (2007), Lundstam et al. (2015), Lundstam et al. (2017), Pretorius et al. (2022)) that featured a similar patient cohort. Despite the overlap in the patient population, these studies reported distinct outcomes, thus enriching our study with a plethora of unique data points, including laboratory parameters, baseline bone mineral densities (BMDs), final follow-up body mass index (BMI), the incidence of bone fractures and nephrolithiasis, the number of patients who initially underwent parathyroidectomy but were later observed, and mortality rates [13,16,17,18]. The utilization of non-overlapping patient populations from these studies resulted in a total sample size of 1346 patients. The presence of overlapping patient populations in these studies might have impacted statistical values such as age, sex, and BMI. However, we decided to include these overlapping studies as they bolstered the statistical power of our study and did not compromise the unique and robust analyses of the outcomes of interest.

The characteristics of eligible studies and that of the primary patient population in our study are shown in Table 1. The average follow-up for all patients in our patient cohort was 41 months, with a standard deviation of 23.8 months. The studies span from 2003 to 2022 and encompass both randomized controlled trials and retrospective and prospective studies. The sample sizes of the studies range from 18 to 216 patients, with female representation varying from 77.8% to 100%. The mean age of participants across the studies fluctuates between 57.5 and 68.2 years, and the follow-up periods extend from 6 to 72 months. In some studies, certain data, such as age or follow-up period, were not reported.

### 3.2. Characteristics of the Study Population

In our endeavor to understand the characteristics of the study population, we focused our analysis on only one of the overlapping studies—Pretorius et al. (2022) [13]. This study encapsulated a total of 865 patients, divided into two distinct cohorts. The first cohort, referred to as the medical group, consisted of 391 patients with an average age of 65.12 years (95% CI = 64.28–65.96). On the other hand, the second cohort, composed of 474 patients who underwent parathyroidectomy, demonstrated a slightly lower average age of 62.56 years (95% CI = 61.77–63.34). Interestingly, the average BMI, a critical determinant of health status, was nearly identical between the two cohorts. Specifically, the average BMI for the non-operative group was calculated to be 29.14 Kg/m^2^ (95% CI = 27.37–30.91), while for the parathyroidectomy group, it was 28.16 Kg/m^2^ (95% CI = 26.90–29.42), a negligible mean difference of only 0.70. Furthermore, the gender composition was also remarkably similar between the two groups, with females constituting approximately 86% of both cohorts, Table 2.

### 3.3. Calcium Levels

Regarding the biochemical analysis, we compared the serum calcium levels of the 632 patients who underwent medical management with the 714 patients who underwent parathyroidectomy. The baseline average serum calcium was found to be similar between the two groups with 10.58 mg/dL (95% CI = 10.54–10.63) in the medically managed group and 10.68 mg/dL (95% CI = 10.63–10.73) in the parathyroidectomy group (*p* = 0.15). The final calcium was similar to the baseline calcium in the medical management group at 10.46 mg/dL (95% CI = 10.39–10.54); however, the post-operative final calcium for the parathyroidectomy cohort was 9.39 mg/dL (95% CI = 9.27–9.52). The final serum calcium was significantly higher in the medical management group as compared to those receiving a parathyroidectomy (*p* < 0.01). These results are summarized in Table 3.

### 3.4. Parathyroid Hormone (PTH) Levels

PTH levels also exhibited a stark contrast between the two groups. While the baseline PTH was slightly higher in the parathyroidectomy group (98.17 pmol/L, 95% CI = 83.19–113.15) compared to the medically managed group (83.84 pmol/L, 95% CI = 80.06–87.63, *p* = 0.06), the final PTH level showed a significant surge in the medically managed group (106.14 pmol/L, 95% CI = 94.44–117.85) and a substantial reduction in the parathyroidectomy group (43.25 pmol/L, 95% CI = 39.03–47.47), *p* = 0.001. These results are summarized in Table 3.

### 3.5. Bone Mineral Density (BMD) Measurements

Upon conducting a meticulous examination of the BMD measurements, it was discerned that the mean baseline total body BMD exhibited no significant disparities between the medically managed group and the parathyroidectomy group. Specifically, the total body BMD was consistent across the board with a SMD of 0.07 and a CI ranging from 0.24 to 0.38 (*p* = 0.54). Delving further into the specific areas of the body, the lumbar spine BMD displayed a slightly negative SMD of −0.05, with the 95% CI stretching from −0.22 to 0.12 (*p* = 0.72). Femoral BMD showcased an even slighter negative SMD of −0.01 with a 95% CI from −0.20 to 0.17 (*p* = 0.61). The hip BMD, on the other hand, exhibited a minuscule positive SMD of 0.01 with a 95% CI from −0.53 to 0.55 (*p* = 0.05). Lastly, the forearm BMD presented a slightly positive SMD of 0.05 with a 95% CI ranging from −0.14 to 0.23 (*p* = 0.16).

**Figure 2:**
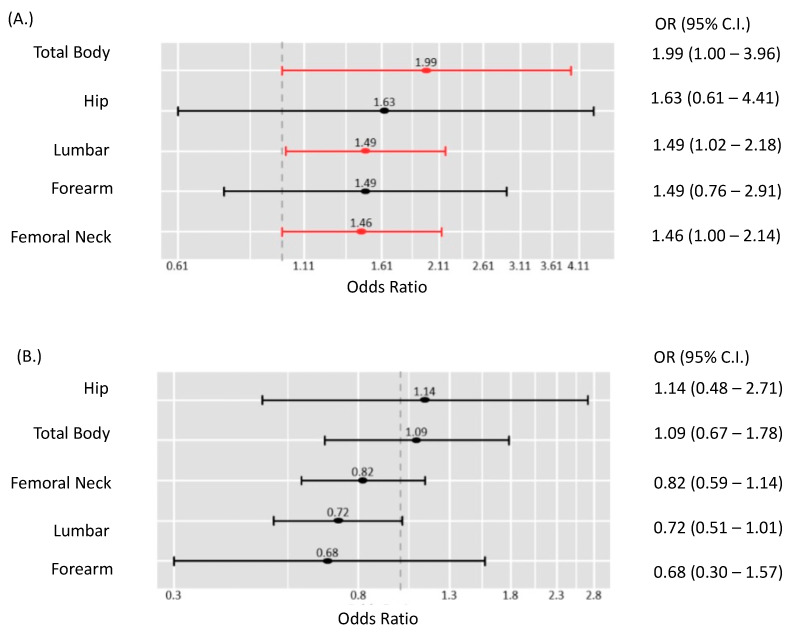
Risk of decrease in bone mineral density after treatment compared to baseline level. (**A**) In the medical group. (**B**) In the surgery group. Standardized mean difference was estimated for pairwise comparison followed by conversion to odds ratio and confidence interval. Pooled estimates of the overall BMD and sub-grouped by anatomical location are represented by horizontal error bars. Red: significantly high risk. Black: no significant risk.

Interestingly, Figure 2A demonstrates that the medically managed cohort was more inclined towards experiencing a decrease in overall BMD over time, perhaps through medication usage, with an OR of 1.99 and a 95% CI ranging from 1 to 3.96. This trend was particularly noticeable in the lumbar and femoral regions. In contrast, Figure 2B underscores that the BMD measurements for the surgical group did not undergo significant alterations.

### 3.6. Complications and Mortality

In terms of complications, the study highlighted no notable differences between the medical and surgery groups. The nephrolithiasis risk was comparable (RR = 2.08, 95% CI = 0.60–7.30). Similarly, there was no difference in overall fracture risk (RR = 1.2, 95% CI = 0.53–2.75), non-vertebral fracture risk (RR = 1.22, 95% CI = 0.58–2.59), or vertebral fracture (RR = 1.37, 95% CI = 0.69–2.72) in the medical management group as compared to the parathyroidectomy group. While limited to only Pretorius et. al., there was found to be no difference in risk for cardiovascular death (RR = 0.99, 95% CI = 0.43–2.27) or overall mortality (RR = 0.87, 95% CI = 0.33–2.29) in the medical management group as compared to the parathyroidectomy group. However, it is crucial to underline that these results hinge significantly on long-term follow-up. Regrettably, our patient population was only monitored for an approximate duration of 42 months, which could potentially limit the scope of these findings. These results are summarized in Table 4.

## 4. Discussion

pHPT, a prevalent endocrine aberration, is escalating in incidence. A substantial portion of all patients diagnosed with pHPT exhibits a milder form of the disorder, which has emerged as the predominant etiology of hypercalcemia in ambulatory care settings [27]. Characteristically, individuals with pHPT exhibit the biochemical hallmark of hypercalcemia, a consequence of excessive parathyroid hormone (PTH) secretion from one or more hyperactive parathyroid glands [8]. The conventional diagnostic criteria for pHPT entails elevated levels of both corrected serum calcium and PTH. This sharply contrasts with the diagnosis of mild pHPT, wherein patients persistently display hypercalcemia, concomitant with PTH levels that hover within the upper-normal range [28,29]. Given the paramount importance of calcium homeostasis in maintaining optimal cellular functionality, enduring hypercalcemia resulting from mild pHPT has been demonstrated to exert deleterious long-term impacts on skeletal, renal, and psychogenic systems [16,20]. As such, an essential objective of treatment should be to orchestrate the stabilization of the biochemical milieu in patients with mild pHPT. Multiple therapeutic avenues are available for individuals afflicted with mild pHPT, spanning from either medical management to surgical intervention. The present discourse endeavors to provide a comprehensive and current meta-analysis, meticulously dissecting the outcomes associated with these divergent treatment modalities.

Our comprehensive meta-analysis elucidated that post-therapeutic calcium concentrations were markedly elevated in the patient cohort managed medically. Intriguingly, post-treatment calcium levels in the medically managed patients mirrored those observed at the commencement of treatment. This inference suggests that the medical management of hypercalcemia in the context of mild pHPT may be somewhat ineffective, although this might be attributable to extraneous factors such as patient non-adherence. In the majority of cases (greater than 95%), parathyroidectomy promptly and effectively mitigates both calcium and PTH levels, resulting in postoperative values that are significantly lower than preoperative levels [30]. Consequently, patients managed medically might be exposed to a heightened long-term risk pertaining to their skeletal, renal, and psychogenic systems.

Persistent hypercalcemia exerts deleterious effects on the musculoskeletal system. Specifically, hypercalcemia detrimentally affects both cortical and trabecular bone development and maintenance, thereby compromising bone health and escalating the risk of fractures [31,32]. Several works have reported that mild pHPT and classic pHPT patients develop fractures and osteoporosis at similar rates [33,34]. In our study, patients undergoing medical management had lower lumbar, femoral, and hip BMD as compared to those managed by parathyroidectomy. Similarly, total body bone mineral density also displayed a declining trend. While the incidence of fractures did not exhibit a significant discrepancy between our two study cohorts, our study was somewhat constrained by a mean follow-up duration of only 42 months (approximately 3.5 years), which inhibited an accurate long-term comparison. An investigation assessing fracture risk in hypercalcemic patients uncovered a 30% surge in the incidence of fractures at the 15-year mark post-surgery [35]. Furthermore, a study by Ramos et al. reported fracture rates of 9.8% (*N* = 11/112) and 13.8% (*N* = 8/58) in the cohorts treated surgically and non-surgically, respectively, over a 2–6-year follow-up period (*p* = 0.436) [21].

Hypercalcemia can precipitate renal dysfunction, typically manifesting as nephrocalcinosis, nephrolithiasis, and hypercalciuria. Given the increased filtered load of calcium in patients with mild pHPT which surpasses the reabsorption capacity of the renal tubular system, hypercalciuria is observed in approximately one-third of mild pHPT cases [36]. A propensity score matching study juxtaposing patients with mild pHPT (*N* = 1424) and a normocalcemic cohort (*N* = 7120) revealed that patients with mild pHPT were disproportionately predisposed to renal failure by a staggering 14 fold (HR = 13.83, 10.41–18.37, *p* < 0.001), with a 5-fold increased likelihood to develop renal stones (HR = 5.15, 2.69–9.83, *p* < 0.001), and were almost twice as likely to develop osteoporotic fractures (HR = 1.63, 1.22–2.19, *p* < 0.001) [37]. This prior finding suggests that surgical treatment for mild pHPT is a logically far superior option, as opposed to medically managed alternatives. Alas, the previously described findings on renal function are contradicted by more recently published randomized control trials [16,25,38]. While our study did not assess for end-point renal failure, this controversy provides the basis for further analysis of eGFR when assessing surgical vs. non-surgical options for the management of mild pHPT. Moreover, though nephrolithiasis is noted on presentation nearly 18% of the time and calcium-based stones are the most common form, our investigation did not discern a significant difference in the reported incidence of nephrolithiasis between patients opting for medical management or parathyroidectomy for their mild pHPT [32,39]. This could be partially attributed to the protracted timeline for kidney stone formation, necessitating a more extended follow-up duration for a robust comparison. Additionally, assessing renal function with precision can be challenging, given its deterioration with age and under the influence of myriad factors [40]. It is worth noting that the prevalence of renal stones in mild pHPT patients, often asymptomatic and detected only on imaging studies, is reported to range between 7–15%, and consequently present without complaint [41]. There is an emerging perspective that suggests that the introduction of an algorithm in the treatment plan that includes regular renal imaging could prove beneficial. This could help identify patients with silent nephrolithiasis, thereby enabling early intervention and possibly preventing the development of more severe renal complications. Further research is needed to validate this hypothesis and to determine the most effective imaging methods and frequency of screenings.

Furthermore, patients with mild pHPT frequently experience neuropsychiatric symptoms and a diminished quality of life as compared to the general population [16,34]. These patients often present with nebulous symptoms such as mood changes, irritability, fatigue, and memory loss, the biochemical underpinnings of which remain elusive [42]. There are reports suggesting that the near-equivalent rates of fatigue (~60% of patients), nocturia (~40% of patients), and difficulty concentrating (~40% of patients) are similar between patients with calcium levels greater than as well as less than 11.2 mg/dL [43]. Moreover, rates of depression are significantly higher in patients suffering from pHPT (31.4%) as compared to benign thyroid disease (15.3%) [44]. An array of studies, including three randomized controlled trials, reported ameliorated neuropsychiatric symptoms among patients undergoing parathyroidectomy compared to non-operative cohorts [16,23,25]. In 2013, Weber et al. also noted that anxiety, depression, and suicidal ideation all significantly subsided post parathyroidectomy [45]. Consequently, the manifestation of neuropsychiatric symptoms serves as an indication for parathyroidectomy, as this procedure may assuage or altogether resolve these symptoms [5]. Innovative research should focus on unraveling the exact biochemical pathways that link mild pHPT to neuropsychiatric symptoms. Understanding these pathways could pave the way for novel therapeutic approaches that could help manage these symptoms more effectively in patients with mild pHPT, potentially enhancing their quality of life.

Parathyroidectomy stands as the sole definitive treatment for patients with mild pHPT, boasting cure rates exceeding 95%, with reported instances of persistent and recurrent disease hovering at 0.5% and 2.4%, respectively [46]. The utilization of intraoperative adjuncts, such as intraoperative parathyroid hormone (PTH) monitoring and radio-guided parathyroidectomy, considerably contributes to these impressive success rates [47]. However, it is essential to acknowledge that parathyroidectomy, while typically curative, also confers iatrogenic risks, including recurrent laryngeal nerve damage, hematoma formation leading to airway obstruction, and hypoparathyroidism [48,49]. Despite these risks, rates of cure and complications in patients who undergo minimally invasive parathyroidectomy stand at an astounding 99.4% and a comparatively minimal 1.5%, respectively [50]. Still, these risks are obviated in patients electing to undergo medical management. Furthermore, medical management allows patients to undergo treatment without obtaining a transcervical scar, which is an appreciable concern [51]. Remote-access parathyroidectomies, though available, have not gained widespread acceptance globally or even within the United States [52,53,54]. Nevertheless, given the remarkable cure rate and safety profile, parathyroidectomy is generally advocated in patient populations deemed fit for surgery. In vulnerable patient populations, such as those with significant cardiac or pulmonary conditions, or those who might struggle to maintain adherence to daily medication, minimally invasive procedures may be considered such as radiofrequency ablation (RFA). This technique, which has been extensively studied in the realm of thyroidology, uses an electrode to deliver localized heat, affecting the ablation of soft tissue, and has shown impressive efficacy with low complication rates in treating pHPT [55,56,57].

Interestingly, the incidence of the multi-gland disease has been found to be higher in patients with mild pHPT when compared to those with the classical presentation of the disease. Mild pHPT has been discovered to have multi-gland involvement in up to a staggering 50% of cases [8]. A separate study conducted by Tordjman et al. discovered that 18% of patients diagnosed and treated with mild pHPT were found to have hyperplasia and up to 82% to have a single adenoma [58]. Moreover, when comparing pathology, patients with the mild form of pHPT have been found to have lower-weight adenomas and overall smaller amounts of parathyroid tissue than their classical pHPT counterparts [33].

Though both cohorts suffered from mild pHPT, one inherent limitation is that patients who were poor surgical candidates are more likely to undergo medical management treatment. This could potentially bias the results in favor of those who underwent parathyroidectomy. Yet, seven of the included studies were randomized controlled trials which slightly mitigated this limitation. Moreover, another limitation is the definition of mild pHPT, which researchers and clinicians sparsely agree upon. Another limitation is the follow-up time of the study, which was only 42 months on average. This restricts our ability to fully gauge the long-term impacts of each treatment modality on mortality and morbidity. Lastly, the medical approach was not strictly delineated across the studies included and was therefore difficult to assess. Future research endeavors should strive to address these limitations and allow for more extended follow-up periods. Regardless, the substantial sample size and wide geographic distribution provided a robust comparative analysis of the two treatment modalities, ensuring generalizability.

## 5. Conclusions

In conclusion, our meta-analysis strongly suggests that parathyroidectomy surpasses medical management in treating mild pHPT patients in terms of biochemical parameters and skeletal health. It emphasizes the pressing need for more studies scrutinizing the long-term outcomes of parathyroidectomy in patients with mild pHPT. The comprehensive understanding of the benefits and risks associated with each treatment approach will enable a more nuanced, patient-centered decision-making process, ultimately improving patient outcomes in this increasingly common neuroendocrine disorder.

## Figures and Tables

**Table 1:** Characteristics of eligible studies and their patient populations.

Author	Year	Design	Sample Size	Female, %	Age, Years (mean ± SD)	Follow-Up, Month (mean)
Pretorius et al. [13]	2022	RCT	191	86.4	63.1 ± 7.8	60
Tzikos et al. [19]	2021	Prospective	38	89.5	NR	36
Khan et al. [20]	2021	Retrospective	60	85.0	68.2 ± 8.9	NR
Ramos et al. [21]	2019	Retrospective	170	92.4	64.6 ± 11.6	72
Lundstam et al. [18]	2017	RCT	145	86.9	62.5 ± 7.5	60
Lundstam et al. [17]	2015	RCT	145	86.9	62.5 ± 7.5	60
Perrier et al. [22]	2009	RCT	18	83.3	63 ± 17	6
Bollerslev et al. [16]	2007	RCT	191	86.4	64.1 ± 7.4	24
Ambrogini et al. [23]	2007	RCT	50	92.0	64.5 ± 6	12
Hagström et al. [24]	2006	Prospective	69	100.0	65.9 ± 5.7	60
Rao et al. [25]	2004	RCT	53	79.2	64.9 ± 7	12
Rao et al. [26]	2003	Retrospective	216	77.8	57.5 ± 11	49

RCT = randomized control trial, NR = not reported.

**Table 2:** Demographics pooled estimates of single-arm meta-analysis for mild pHPT patients.

Demographic Data	Group	Studies	Effect Size		Heterogeneity	
			Estimate	95% CI	I^2^	*p*-Value
Age, years	Medical	9	65.12	64.28–65.96	93%	<0.01 *
	Surgery	9	62.56	61.77–63.34	92%	<0.01 *
Sex: (female)	Medical	9	86%	80–91%	42%	0.09 $
	Surgery	9	86%	90–91%	39%	0.11 $
BMI, kg/m^2^	Medical	5	29.14	27.37–30.91	64%	0.03 *
	Surgery	5	28.16	26.90–29.42	62%	0.03 *

* Random effects model was used due to significant heterogeneity. Raw mean estimates are reported. $ Arcsine transformed proportion was reported. CI: confidence interval.

**Table 3:** Biochemical pooled estimates of single-arm meta-analysis for mild pHPT patients.

Biochemical Data	Group	Studies	Effect Size		Heterogeneity	
			Estimate	95% CI	I^2^	*p*-Value
Baseline calcium (mg/dL)	Medical	6	10.58	10.54–10.63	95%	<0.01
	Surgery	6	10.68	10.63–10.73	94%	<0.01
Post-treatment calcium (mg/dL)	Medical	4	10.46	10.39–10.54	92%	<0.01
	Surgery	5	9.39	9.27–9.52	80%	<0.01
Baseline PTH (pmol/L)	Medical	8	83.84	80.06–87.63	94%	<0.01
	Surgery	8	98.17	83.19–113.15	94%	<0.01
Post-treatment PTH (pmol/L)	Medical	5	106.14	94.44–117.85	72%	<0.01
	Surgery	6	43.25	39.03–47.47	74%	<0.01

Data are reported as raw mean estimates. CI: confidence interval. Random effects model was used for all parameters.

**Table 4:** Pairwise comparison for risk of complications in mild pHPT patients.

Complications	Studies	Effect Size		Heterogeneity	
		RR	95% CI	I^2^	*p*-Value
Nephrolithiasis	3	2.08	0.60–7.30	0%	0.84
All fractures	3	1.20	0.53–2.75	91%	<0.01
Non-vertebral fracture	3	1.22	0.58–2.59	81%	<0.01
Vertebral fracture	3	1.37	0.69–2.72	42%	0.18
Cardiovascular death	1	0.99	0.43–2.27	NA	NA
Disease-related mortality	1	0.87	0.33–2.29	NA	NA

RR: relative risk, CI: confidence interval.
